# Confronting diversity in the production of clinical evidence goes beyond merely including under-represented groups in clinical trials

**DOI:** 10.1186/1745-6215-14-177

**Published:** 2013-06-15

**Authors:** Karien Stronks, Nicolien F Wieringa, Anita Hardon

**Affiliations:** 1 Department of Public Health, Academic Medical Center, University of Amsterdam, Amsterdam, The Netherlands; 2 Communication Department, University Medical Center Groningen, University of Groningen, Groningen, The Netherlands; 3 Amsterdam Institute for Social Science Research, University of Amsterdam, Amsterdam, The Netherlands

**Keywords:** Evidence-based medicine, Diversity, Clinical trials

## Abstract

There is increasing evidence that outcomes of health care differ by patient characteristics, such as gender and ethnicity. If evidence-based medicine is to improve quality of care for all patients, it is essential to take this diversity into account when designing clinical studies. So far, this notion has mainly been translated into recommendations for including minority populations in trials. We argue that a more comprehensive view of the production of diversity-sensitive clinical evidence is needed, one that takes heterogeneity as a starting point in research. We call for a mix of methodological approaches aimed at identifying diversity issues that matter and analysing the impact of these diversities on clinical outcomes. Institutional changes are necessary to support this methodological reform.

## Introduction

Evidence-based medicine (EBM) is the conscientious, explicit, and judicious use of current best evidence in making decisions about the care of individual patients [1]. The main aim has been to ground medical decision-making in high-quality evidence, with the ultimate aim of improving quality of care. Randomized controlled trials (RCTs) that show whether a treatment is efficacious represent the gold standard in EBM. Originally, RCTs were thought to produce knowledge applicable to all patients. More recently, this assumption has been criticized [2-4]. There is increasing evidence that treatment outcomes as well as disease progression and manifestation might vary between patient groups. Examples include the different way that black patients respond to specific antihypertensive drugs [5], and sex differences in the epidemiology of heart disease [6]. If EBM is to improve quality of care for all patients, when designing clinical studies it is essential to consider diversity in the efficacy of treatments and the aetiology and manifestation of diseases. Diversity in clinical research is thus a prerequisite for equity in health [7,8].

Regulatory reforms in the mid-1990s, particularly those in the USA, drew attention to this issue [4]. These regulatory reforms appear to have led mainly to greater representation of women, the elderly, and ethnic minorities in trial populations. In fact, a whole new field of study has developed, labelled by Epstein [9] as ‘recruitmentology’: empirical studies that evaluate the effectiveness of recruitment strategies for including people from minority populations. An example of this is the Eliminating Disparities in Clinical Trials (EDICT) Project, which aimed to develop and implement policy solutions to promote the inclusion of minority populations in clinical trials [10]. This resulted in a broad range of recommendations, from professional education to community involvement in the process of designing a clinical trial.

However, only some of these studies have the potential for subgroup analysis, which is a prerequisite for assessing differences in treatment outcomes between groups. For example, after looking at 86 original articles in medical journals that reported on clinical trials funded by the National Institutes of Health (NIH), Geller *et al*. [11] concluded in 2011 that only 25% of the studies reported sex-specific results, and less than half of the studies analysed the results by ethnic group. In most cases, the ethnic minority groups were too small to allow for subgroup analysis. In fact, the authors found very few improvements when compared with their previous analysis five years earlier. As a result, 20 years after the introduction of regulatory reforms calling for analysis by age, sex, and ethnicity, there is still little evidence on diversity in the efficacy of treatment, leading to a lack of diversity-sensitive guidelines for professionals.

The challenges are great. A multidisciplinary project commissioned by the Netherlands Organisation for Health Research and Development (ZonMw) - involving clinicians, epidemiologists, ethicists, sociologists, and anthropologists - examined factors that both facilitate and constrain a focus on diversity in clinical research. The project included a number of literature reviews, and a conference in which the conclusions of these reviews were discussed [12]. Based on this project, in this paper we put forward recommendations on the methodology and institutional mechanisms that can facilitate a systematic and comprehensive production of diversity-sensitive evidence. Before presenting these recommendations, we will first make the case for the importance of having a comprehensive view of diversity that goes beyond merely including minority populations in clinical trials.

## Review

### Framing the issue of diversity in the production of clinical evidence

From an epistemological view, homogeneity is the dominant paradigm in clinical research. This is apparent from the methodology of clinical trials: strict inclusion and exclusion criteria are set for clinical trials to minimize the chance of side effects, comorbidity, and early dropout [13]. Homogeneity of the population to be studied is considered important, as it improves a trial’s internal validity and statistical power [13].

Consequently, diversity issues are frequently framed as deviations from the homogeneous population. Given the under-representation of ethnic minorities, women, and older people, this generally means that these are deviations from the white male population. But this does not mean that diverse groups (as defined by sex, ethnicity, or other background characteristics) are always different from each other with respect to health or medical care. On the contrary, the literature contains many examples of studies showing that the effects of clinical interventions do not differ between subgroups. Well-known examples include the use of aspirin to prevent coronary heart disease, which is now thought to be as effective in women as in men, despite the results of previous trials pointing to its ineffectiveness in women [14,15].

However, deliberating about populations that may or may not differ from the standard population will in itself not be enough to ensure a thorough understanding of inequalities between populations. The limitations of this approach are twofold. First, it looks at diversity issues from the perspective of the standard only (such as ‘male patients’). For example, this has led to an emphasis on the specific health risks faced by women (related to their different reproductive system, et cetera.). In this perspective, the specific health risks faced by men are likely to be neglected. If we take gender sensitivity rather than deviations from the male norm as a starting point for analysis, this might draw our attention to characteristics such as the attitudes and values that underlie differences between genders, including masculinity and caring capacities [16,17]. Second, background characteristics such as age, sex, and ethnicity do not in themselves have aetiological consequences. In defining diversity issues that matter, studies should in fact consider a broad range of relevant factors, including biological, genetic, sociocultural, psychological, economic, and behavioural factors which may have impact on the efficacy of health care services and the health of individuals. This exploration goes far beyond the influences of variables that have traditionally been used to distinguish between groups of patients (such as ‘ethnicity’), which function as surrogate classifiers of relevant biomedical and sociocultural differences [18]. For example, in osteoporosis, an important sex difference in bone loss is related to the mechanisms of bone deterioration [19]. Thus, studying the efficacy of medication on these different mechanisms rather than the differences between sexes is likely to provide knowledge that is highly relevant to the treatment for various patient groups. A more nuanced understanding of differences and similarities between people is thus likely to address the underlying biological and/or sociocultural mechanisms.

If we are to take the principle of diversity-sensitive clinical knowledge seriously, it needs to be made the core perspective in study design, starting with the conceptualization of the problem to be studied. This requires that the hypotheses underlying studies based on a nuanced understanding of possible differences between groups should be studied in a population that represents groups that differ on the aspects considered to be relevant. This will help to produce clinical knowledge that can be applied to the entire population, as it allows for a detailed understanding of why a specific intervention works and for whom. As such, producing diversity-sensitive clinical knowledge is also likely to benefit the standard population to which trials are usually restricted.

### Towards a methodological reform

What are the implications for clinical research methodology if we take heterogeneity as the guiding principle?

RCTs can, of course, be used to study heterogeneity. If the effect of the intervention under study is hypothesized to differ between subgroups, such as the hypothesis that the preventive effect of aspirin differs between men and women, there are two options. First, new RCTs can be designed that specifically test treatment effects in relevant subgroups, as was done in the Women’s Health Initiative, which critically examined the existing evidence on the preventive effects of hormone replacement therapy on cardiovascular disease [20]. A second option is to enlarge the original trial with sufficient members from each subgroup, so that the potential differential effect - called effect modification in epidemiological terms - can be studied. This implies the a priori creation of a subgroup, driven by specific hypotheses as to differences in treatment effect. In addition, the subgroups should be appropriately powered to detect the hypothesized effect size difference [21].

When aiming to include subgroups that are traditionally under-represented in clinical trials, many problems might be encountered in terms of adequate trial enrolment, the validity of measurements (including self-reported measurements) in different groups, and compliance and retention (for example, in ethnic minority groups) [22]. In view of the methodological literature, it appears that the majority of these problems can be overcome in principle. Effective strategies for this are targeted communication, and anticipating the cultural and cognitive characteristics of specific populations [23-26]. To create conditions for heterogeneous study populations, it is essential to further develop instruments and strategies that suit the needs of those minority populations that are difficult to recruit with the available instruments [26].

However, this does not imply that all clinical trials should automatically include subgroups. Ensuring the inclusion of subgroups large enough to allow for subgroup analysis has an enormous impact on costs. In view of limited resources, the choice for a heterogeneous study population should at least be weighed against the strength of the indications for the presence of diversity in health outcomes. This implies that confronting diversity in clinical research starts with formulating hypotheses as to why diversity does or does not matter in a specific case.

To identify the diversity issues that matter for health outcomes, a mix of different methodologies is needed. An increasing number of complementary research methods are available as shown below.

Reanalysis of past trials: researchers can critically review past trials, exploring unexpected phenomena in RCTs and identifying variance in effects, such as outliers. Meta-analysis at an individual level can be used to explore differences in treatment outcomes by subgroup. A meta-analysis on the effects of chemoradiotherapy for cervical cancer in subgroups of women by patient variables could serve as an example [27]. Of course, we acknowledge the weaknesses of subgroup analysis. The most important downside of this strategy is the risk that in the case of multiple subgroup analyses, a difference from the overall results will be found in one or more comparisons even if none exists (type 1 error). However, from an equity perspective, ‘rejecting all such analyses may risk throwing the baby out with the bathwater’, as argued by Petticrew *et al*. [21].

Observational studies: researchers can review the increasing number of biomedical and pharmacodynamic studies that reveal how aetiology, prognosis, disease perception, and/or effects of interventions are affected by age, sex and/or ethnicity and by other factors [28,29], or the intersectionality between these factors [17,30], supported by the development of statistical methods to establish causality [31]. In addition, population-based observational studies can be used to explore a wide range of possible associations between diversity variables and treatment outcome, on their own merits and for generating hypotheses for the relevant RCTs. For example, population studies on the contraceptive pill have identified smoking and age as relevant dimensions of diversity associated with the risk for thrombosis [32]. It should be acknowledged, however, that the problem of under-representation of groups such as ethnic minority populations, as discussed earlier in terms of clinical trials, apply equally to observational studies. For example, in their 2006 review of 72 cardiovascular cohort studies, Ranganathan and Bhopal concluded that only 15 of these were able, by design, to compare different ethnic groups. All of these were performed in the USA. Of the 41 studies in Europe, none was able to provide data by ethnic group [33], which limits their usability for generating diversity hypotheses. Investments are warranted to increase usability so that aetiological and epidemiological knowledge can be produced based on observational studies that also assume heterogeneity.

Databases of routine health care: researchers can consult databases of patients registered with, for example, a general practitioner for routine health care, to explore effect modification in treatment outcomes. Databases of adverse drug reaction reports may also be relevant. An analysis of a population-based birth defect registry in The Netherlands (showing an interaction between maternal smoking and high body mass index for the occurrence of specific congenital heart anomalies in offspring) might serve as an example [34]. In general, these databases allow for only a crude distinction between patient groups, based on variables such as sociodemographic characteristics, comorbidities, and concurrent medicine use. The most important limitation of this strategy, therefore, seems to be the lack of detailed information on patient characteristics that might account for a differential effect of treatment (such as smoking or body mass index in the example given above). These routine databases might nevertheless be a starting point for generating hypotheses.

Qualitative studies: these can also help identify diversity issues that matter for health outcomes. First, qualitative evidence might arise from observations by patients and professionals, as they are the first to observe differences in treatment outcomes. What differences are observed? What causes these differences? Are there puzzling phenomena, unexpected side effects, or unidentified variances in treatment effects? Physicians sometimes publish these valuable experiences as case reports. The experiential knowledge of patients and the clinical experience of health professionals have been shown to complement those of researchers [35]. Second, scientific research that uses qualitative methodology has been increasingly recognized as making an important contribution to EBM. This includes studies that aim to understand why specific interventions tested in a RCT work, as well as studies that generate hypotheses as to the potential differential effect of an intervention to be tested in a RCT [36].

Once the diversity hypotheses have been identified, further analysis and testing is needed. This could lead directly to the recommendation to include subgroups in RCTs, but also to aetiological studies to investigate possible underlying causes of the differences if these are unknown. The development of diversity hypotheses as we propose here might help to compensate for the above-mentioned weakness of the subgroup analysis strategy, that of drawing misleading conclusions: the more precisely the hypotheses as to why groups might differ with regard to the intervention effect are formulated before the data collection, the more likely the study will result in valid estimates of differential effects of the intervention. The subgroups to be involved in the analyses can then be defined so that they have aetiological consequences. For example, in the field of hypertension, if ethnicity has been related to differences in plasma renin levels as an effect modifier, future trials could consider this as the relevant variable rather than ethnicity.

### Institutional changes necessary to support this methodological reform

The production of diversity-sensitive clinical knowledge also requires institutional mechanisms and arrangements that facilitate the methodological reform described above. Although the role of funding agencies seems to be crucial in this respect, we would like to point out the responsibilities of other organizations as well.

#### Funding agencies

The NIH in the USA seems to have been most successful in implementing specific diversity-relevant programmes. Within the organizational structure of the NIH, offices can be established to increase research on specific subjects. To stimulate research on women’s health issues, the NIH established the Office of Research on Women’s Health (ORWH) in 1990. Other examples include the National Institute on Minority Health and Health Disparities (NIMHD) at the NIH [4]. In Europe, the focus has been on mainstreaming rather than on specific programmes. For example, as a follow up to a gender assessment that was part of the Fifth Framework Programme (FP5), that noted that projects addressing sex/gender differences did so in a very limited way, the European Union (EU) has commissioned the development of manuals for scientific and project officers to provide them with guidance on how to implement gender mainstreaming throughout the entire process of funding research [37].

If diversity-relevant research is mainstreamed, funding organizations should facilitate attention for this issue throughout all working processes. This includes the processes by which relevant committees review funding proposals. At this stage, attention for diversity issues can be facilitated by ensuring that all proposals include a concise and systematic review of the relevant evidence on diversity, that they specify how diversity in disease aetiology, progression and manifestations, and treatment outcomes has informed the study design, and that they specify which of the other diversity issues that matter will be analysed in the study.

The way diversity is conceptualized is crucial in this respect. Both the NIH and the EU have chosen to focus on broad subgroups, such as women or older people. In line with our methodological recommendations, additional dimensions of diversity require such mainstreaming. In addition, there is a need for calls for proposals that allow for multidisciplinary and multi-stakeholder programmes aimed at producing a broader range of diversity-sensitive clinical evidence, which is still lacking at present. These programmes should include funding for innovative studies that use a mix of qualitative and quantitative methods, and which aim at generating and analysing diversity-relevant hypotheses. The funding programmes should be committed to further analyses and testing once relevant diversity issues have been identified, with the overall aim of producing better evidence on diversity to guide medical practice.

Funding agencies in several countries have also developed guidance documents for researchers to enhance the quality of research among subgroups, such as minority populations. The effectiveness of this kind of guidance, which contains methodological and practical recommendations, has not been studied widely. Recent experiences from a major funder of social science research in the UK suggest that this guidance has little impact on practice. The authors conclude that in order to have an impact, this guidance needs to be promoted more vigorously [38].

#### Other stakeholders

Apart from funding agencies, other stakeholders also seem to be vitally important. These include (but are not limited to) researchers, professional organizations, patient organizations, and medical ethics committees.

Researchers should design studies more carefully to allow for the analysis of diversity issues that matter, as described in the first part of this paper. In determining endpoints for their studies, they need to more systematically consider diversity in aetiology as well as manifestation and progression of disease. In defining diversity issues that matter, they should in fact consider all relevant factors that may influence the efficacy of health care.

The professional organizations of general practitioners and specialists tend to identify gaps in diversity-sensitive evidence when developing treatment guidelines. The gaps can guide research programmes. They can also help to identify the underlying philosophical assumptions of the observations of current research, as well as generate hypotheses on diversity issues that matter from clinical observations.

Patient organizations can also play a key role in identifying diversity issues. Funding agencies increasingly have patient representatives on their committees, which often raises issues about representation. Who can be considered to represent a specific patient population or community? The position developed by INVOLVE, a British advisory board that supports greater public involvement in health research, is that involvement of patients or consumers should not aim to represent users, but to seek different perspectives: involving a range of people introduces a range of perspectives [39]. Although consumer involvement in the design of trials (such as in the UK) has increased over time, most trials still have no such involvement [40]. In addition, although researchers feel that the contribution of consumers is worthwhile, there seems to be room for improvement regarding the impact consumers have on the way research questions are being framed, including aspects of diversity [40].

Medical ethics committees can play a key role as well [41]. Historically, ethical debates in clinical research have focused on patient protection, expressed mainly as doing no harm, and autonomy in decision making. Increasingly, such committees are considering participation and representation in clinical research. An important issue, therefore, is to consider barriers to participation, how these may differ between patient groups, and how access to research can be achieved.

## Conclusions

The importance of producing diversity-sensitive evidence for the development of guidelines for clinical care has been acknowledged for decades. Solutions have focused mainly on including under-represented groups in trials. Although this issue is still of the utmost importance, the production of diversity-sensitive clinical evidence requires more than just this. We argue that heterogeneity should be the starting point of clinical trials, implying an exploration of diversity issues that matter for the outcome of health care services. To produce the evidence that justifies clinical trials that take diversity issues into account, a mix of hypothesis-generating and hypothesis-testing research is needed, involving studies that use a mixed-methods approach. Confronting diversity in clinical research thus demands a programmatic and iterative approach that ensures an ongoing interest in relevant variance. Stakeholder participation is key. Doctors and patients can alert the researchers to diversity issues that matter in routine health care, thus contributing to a more relevant evidence base for practice. In addition, a broad conceptualization of diversity is crucial in this respect. In analysing diversity in treatment outcomes, the design of RCTs needs to be based on a nuanced understanding of the interplay of the factors underlying patients’ background characteristics, including genetic, environmental, sociocultural, economic, and behavioural factors. This methodological reform can only be achieved if it is supported by institutional changes. Diversity issues need to be mainstreamed into all phases of funding programmes for health research, from commissioning to implementation. In addition, many other actors, including professional organizations and medical ethics committees, should incorporate diversity as a core value in the way they operate.

We hope our reflections will inspire others who take diversity in the production of clinical evidence seriously. This trajectory is essential, so that the further development of EBM will benefit all patients.

## Abbreviations

EBM: Evidence-based medicine; EDICT: Eliminating Disparities in Clinical Trials; EU: European Union; FP5: Fifth Framework Programme; NIH: National Institutes of Health; NIMHD: National Institute on Minority Health and Health Disparities; ORWH: Office of Research on Women’s Health; RCT: Randomized controlled trial; ZonMw: Netherlands Organisation for Health Research and Development.

## Competing interests

The authors declare that they have no competing interests.

## Authors’ contributions

All authors conceived the study, participated in the manuscript design, and performed parts of the literature search. KS and AH drafted the manuscript. NFW critically read and helped to revise the draft. All authors read and approved the final manuscript.
